# Elucidating fungal decomposition of organic matter at sub-micrometer spatial scales using optical photothermal infrared (O-PTIR) microspectroscopy

**DOI:** 10.1128/aem.01489-23

**Published:** 2024-01-30

**Authors:** Michiel Op De Beeck, Carl Troein, Carsten Peterson, Anders Tunlid, Per Persson

**Affiliations:** 1 Centre for Environmental and Climate Science, Lund University, Lund, Sweden; 2 Microbial Ecology, Department of Biology, Lund University, Lund, Sweden; Colorado School of Mines, Golden, Colorado, USA

**Keywords:** microenvironment, microorganism, infrared microspectroscopy, decomposition, organic matter, cellulose

## Abstract

In microbiological studies, a common goal is to link environmental factors to microbial activities. Both environmental factors and microbial activities are typically derived from bulk samples. It is becoming increasingly clear that such bulk environmental parameters poorly represent the microscale environments microorganisms experience. Using infrared (IR) microspectroscopy, the spatial distribution of chemical compound classes can be visualized, making it a useful tool for studying the interactions between microbial cells and their microenvironments. The spatial resolution of conventional IR microspectroscopy has been limited by the diffraction limit of IR light. The recent development of optical photothermal infrared (O-PTIR) microspectroscopy has pushed the spatial resolution of IR microspectroscopy beyond this diffraction limit, allowing the distribution of chemical compound classes to be visualized at sub-micrometer spatial scales. To examine the potential and limitations of O-PTIR microspectroscopy to probe the interactions between fungal cells and their immediate environments, we imaged the decomposition of cellulose films by cells of the ectomycorrhizal fungus *Paxillus involutus* and compared O-PTIR results using conventional IR microspectroscopy. Whereas the data collected with conventional IR microspectroscopy indicated that *P. involutus* has only a very limited ability to decompose cellulose films, O-PTIR data suggested that the ability of *P. involutus* to decompose cellulose was substantial. Moreover, the O-PTIR method enabled the identification of a zone located outside the fungal hyphae where the cellulose was decomposed by oxidation. We conclude that O-PTIR can provide valuable new insights into the abilities and mechanisms by which microorganisms interact with their surrounding environments.

**IMPORTANCE:**

Infrared (IR) microspectroscopy allows the spatial distribution of chemical compound classes to be visualized. The use of conventional IR microspectroscopy in microbiological studies has been restricted by limited spatial resolution. Recent developments in laser technology have enabled a new class of IR microspectroscopy instruments to be developed, pushing the spatial resolution beyond the diffraction limit of IR light to approximately 500 nm. This improved spatial resolution now allows microscopic observations of changes in chemical compounds to be made, making IR microspectroscopy a useful tool to investigate microscale changes in chemistry that are caused by microbial activity. We show these new possibilities using optical photothermal infrared microspectroscopy to visualize the changes in cellulose substrates caused by oxidation by the ectomycorrhizal fungus *Paxillus involutus* at the interface between individual fungal hyphae and cellulose substrates.

## INTRODUCTION

A long-standing goal in microbiology has been to link environmental factors such as pH, temperature, moisture, nutrient concentrations, etc., to microbial processes such as organic matter decomposition. Traditionally, both environmental factors and decomposition activities have been derived from bulk samples. However, a growing body of evidence indicates that this traditional bulk approach is limited in its ability to link environmental factors to microbial processes quantitatively. Consequently, the need for microbial processes to be studied at scales that are much more relevant to the microorganisms involved has been advocated (1).

Infrared (IR) microspectroscopy is a very suitable technique for investigating the spatial distribution of chemical compound classes and is thus particularly useful for mechanistic studies on the modifications of organic substrates by microorganisms. IR microspectroscopy has the advantage that samples can be held under ambient conditions. No vacuum is required, as is the case for many other chemical imaging techniques, such as scanning transmission X-ray spectroscopy, scanning electron or transmission electron microscopy with energy dispersive spectroscopy, X-ray photoelectron spectroscopy, X-ray fluorescence spectroscopy, or nanoscale secondary ion mass spectrometry. IR microscopes are also typically compact bench-top instruments that are more readily available than these other chemical imaging techniques. In addition, unlike fluorescence microscopy, IR microspectroscopy is a label-free technique.

**Fig 1:**
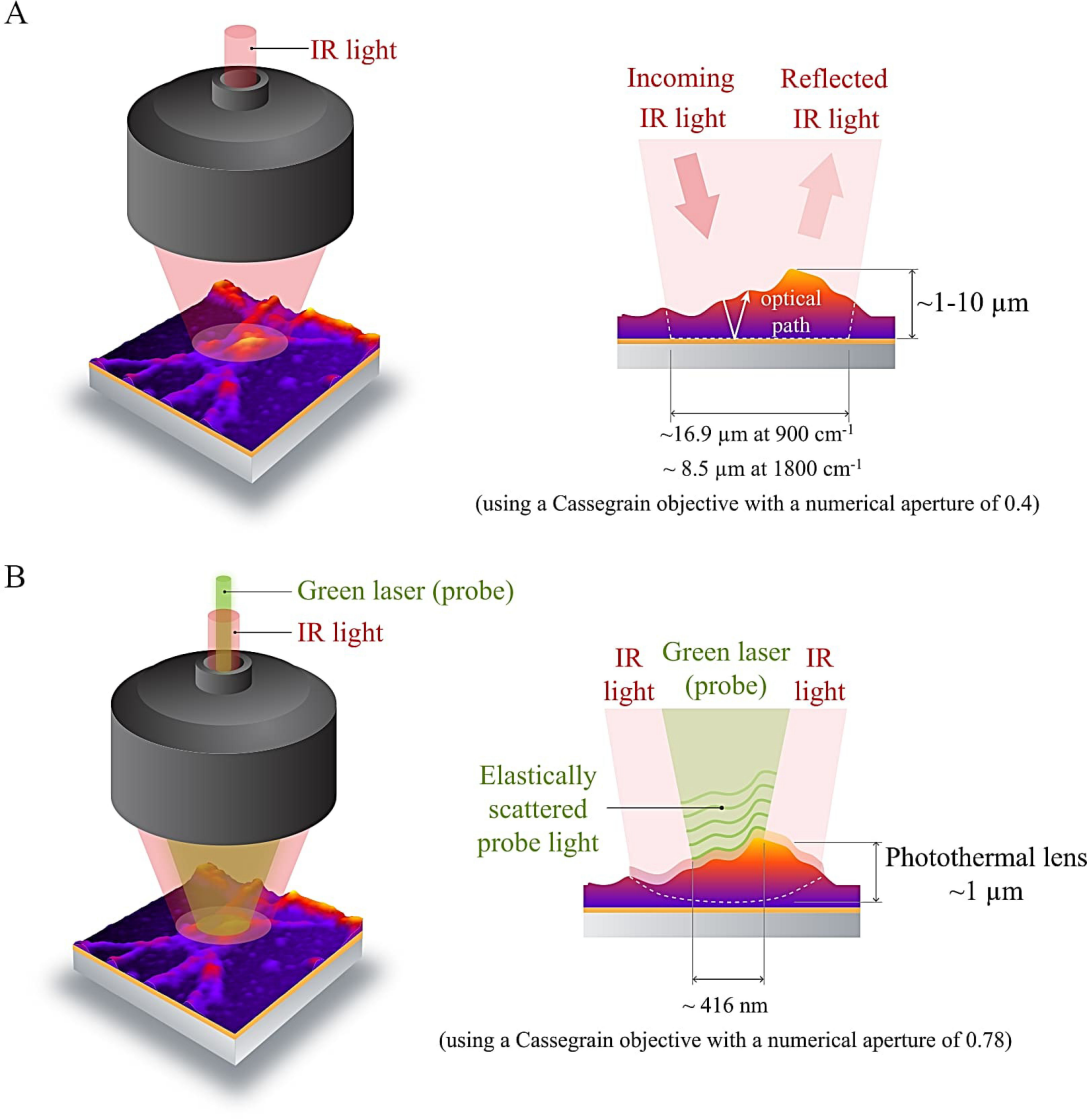
Concept of IR microspectroscopy. (A) Conventional IR microspectroscopy. In conventional IR microspectroscopy, IR light is typically generated by a Globar IR source, which is directed onto a sample through a set of mirrors and a Cassegrain objective in a microscope. In reflection mode, incident IR light passes through a sample and reflects off of a reflective surface (in our study, this was a gold mirror layer on the microscope slide). IR light that has not been absorbed by the sample is then collected on a detector, and the difference between the incoming IR light and the non-absorbed IR light is compared to calculate an absorbance spectrum. The thickness of a sample that is suitable for conventional IR microspectroscopy depends on the optical density of the sample. For biological samples, the sample thickness typically can vary between 1 and 10 µm. The area that is probed by IR light in this set-up is wavelength dependent with a probe area with a diameter of 16.9 µm at 900 cm^−1^ and 8.5 µm at 1,800 cm^−1^, when using a Cassegrain objective with a numerical aperture of 0.4. (B) O-PTIR microspectroscopy. In O-PTIR, IR light is generated by a set of Quantum Cascade Laser chips. This IR light is guided onto a sample through a set of mirrors and a Cassegrain objective in a microscope. The same surface area of the sample is illuminated by IR light as is the case in conventional IR microspectroscopy. Absorption of the IR light by approximately the top 1 µm of the sample generates a photothermal lens. This photothermal lens is probed by a green probe laser, which is made coaxial and confocal with the IR light. In O-PTIR, it is the elastically scattered and reflected green laser light that scatters and reflects off of the photothermal lens that is collected onto a photodiode detector. The changes in intensity in the reflected or scattered green light are proportional to the expansions of the sample caused by IR irradiation, which is used to calculate IR spectra. As a result, the surface area probed in O-PTIR depends on the spot size of the green laser, rather than the spot size of the IR light. In our case, the spot size of the green laser was 416 nm, as we were using a Cassegrain objective with a numerical aperture of 0.78 and a green laser with a wavelength of 532 nm.

Historically, the spatial resolution of IR microspectroscopy, however, has been limited by the diffraction limit of IR light. In such conventional IR microspectroscopy set-ups, typically a Globar light source is used to generate IR light, and a pyroelectric or photoconductive detector is used to detect this IR light. The spatial resolution in this type of IR microspectroscopy technique is wavelength-dependent, with a spatial resolution of 16.9 µm at 900 cm^−1^ to 8.5 µm at 1,800 cm^−1^ (using a Cassegrain objective with a numerical aperture of 0.4) (Fig. 1A). The development of Quantum Cascade Lasers (QCL) (2) and their implementation in IR microspectroscopy in a new technique called optical photothermal IR (O-PTIR) microspectroscopy has pushed the spatial resolution of IR microspectroscopy beyond the diffraction limit of IR light. In O-PTIR microspectroscopy, the thermal expansions in a sample that are generated by the QCL are probed by a green laser (532 nm) and a photodiode. As a result, the spot size that is probed with O-PTIR is 416 nm (using a Cassegrain objective with a numerical aperture of 0.78) (Fig. 1B). O-PTIR thus features a 30 times higher spatial resolution compared to conventional IR microspectroscopy, making it a very suitable technique to look at the interactions between individual microbial cells and their microenvironments.

To examine the potential and limitations of O-PTIR microspectroscopy to probe the interactions between fungal cells and their immediate microenvironments, we imaged the decomposition of cellulose by cells of the ectomycorrhizal fungus *Paxillus involutus* and compared the results obtained using O-PTIR with conventional IR microspectroscopy. Inhabiting the interface between tree roots and soil, ectomycorrhizal fungi are thought to play a key role in soil organic matter dynamics, but their capacity to decompose organic matter derived from plant litter remains unclear (3). *P. involutus* is widespread in the Northern Hemisphere and forms ectomycorrhiza with numerous species of coniferous and deciduous trees, making it an important player in nutrient cycles in Northern Forest ecosystems (4). Moreover, analyses of bulk samples from batch experiments have suggested that *P. involutus* has some capacity to decompose plant cell wall compounds present in soil organic matter using oxidative mechanisms (5, 6).

## MATERIALS AND METHODS

### Preparation of cellulose films

All materials used were of analytical grade.

Gold-coated microscope slides measuring 2.5 cm by 2.5 cm with a 10-nm gold layer on top of a 2-nm titanium adhesion layer were purchased from Platypus Technologies (Madison, Wisconsin, USA). These microscope slides were cleaned by soaking them in 2.5 M NaOH for 1 hour, followed by rinsing with distilled water and MilliQ water. Cleaned microscope slides were dried in an oven at 60°C for 1 hour. After drying, the microscope slides were autoclaved.

Cellulose acetate (product number 419028, purchased from the Merck Group, Darmstadt, Germany) was dissolved in acetone over 24 hours at a weight-to-weight ratio of 8% cellulose acetate to 92% acetone. After dissolving, the solution was filter sterilized using nylon syringe filters with a pore size of 0.45 µm. One microliter of filter-sterilized cellulose acetate solution was then aseptically spin-coated on top of a sterile gold-coated microscope slide at 3,000 rpm for one minute. Spin-coating was performed on a SPIN150i-NPP single substrate spin processor (SPS-Europe B.V., Putten, The Netherlands). The spin coater was placed in a laminar airflow cabinet and sterilized using 70% ethanol.

Microscope slides with cellulose acetate films were subsequently dried for 10 minutes in a laminar airflow cabinet. Slides with films were then submerged in a solution of 0.1 M NaOH in 70% ethanol for 6 hours to remove the acetate sidechains from the cellulose backbone, resulting in regenerated cellulose films. Regenerated cellulose films were finally washed 5 times with 70% ethanol and dried in a laminar airflow cabinet for 1 hour. The complete removal of acetate sidechains from the cellulose films was checked using conventional IR microspectroscopy. Deacetylation was considered complete when the acetate peaks at 1,742 cm^−1^ and 1,219 cm^−1^ were completely removed from cellulose spectra (Fig. S1).

The cellulose films prepared in this way were very flat and thin. They were designed to be suitable for both conventional IR and O-PTIR microspectroscopy. The range of thicknesses a sample can be is much narrower for conventional IR microspectroscopy than it is for O-PTIR. O-PTIR uses the photothermal lens created by the surface of the sample, making the signal obtained relatively independent of the sample thickness. Yet, samples that are too thin will still result in very low signal and poor signal-to-noise ratios. For conventional IR microspectroscopy, on the other hand, the IR light passes through the entire sample (twice in reflection mode, as was the case for this study). As the IR light passes through the sample, some of the IR light is absorbed. Hence, the amount of IR light that is absorbed strongly depends on the sample thickness. Too thick a sample and all IR light will be absorbed, giving no signal. Too thin a sample and not enough of the IR light will be absorbed to give a good signal-to-noise ratio. Also, the flatness of a sample is important. Differences in optical density within a sample can result in Mie scattering in conventional IR microspectroscopy (7). Although artifacts in IR spectra that result from Mie scattering from spherical objects can be corrected rather well (8), Mie scattering caused by more complex structures in samples is much more difficult to correct. O-PTIR spectra are much less affected by Mie scattering, but the way the laser light interacts with different (biological) materials is much less well understood for O-PTIR than it is for conventional IR microspectroscopy. Detailed investigations of possible artifacts in IR spectra generated by O-PTIR will be needed in the future.

### Fungal cultures and growth conditions

Fungal cultures of *P. involutus* (Batsch) Fr. strain ATCC 200175 (Manassas, Virginia, USA) were maintained on solid modified Fries medium (9). The medium contained 406 µM MgSO_4_•7H_2_O, 342 µM NaCl, 1.34 mM KCl, 243 µM H_3_BO_3_, 20 µM ZnSO_4_•7H_2_O, 5 µM CuSO_4_•5H_2_O, 50 µM MnSO_4_•H_2_O, 0.16 µM (NH_4_)_6_Mo_7_O_24_•4H_2_O, 220 µM KH_2_PO_4_, 177 µM CaCl_2_•2H_2_O, 56 µM myo-inositol, 0.30 µM thiamine•HCl, 0.10 µM biotin, 0.59 µM pyridoxine, 0.27 µM riboflavin, 0.82 µM nicotinamide, 0.73 µM p-aminobenzic acid, 0.21 µM calcium-pantothenate, 5.43 mM di-ammonium tartrate, 74 µM FeCl_3_•6H_2_O, 56 mM D-glucose, and 1% wt/vol agar. The pH of the medium was corrected to 4.8. Cultures were grown at 21°C in the dark.

A *P. involutus* culture was grown on solid Fries medium for 7 days. After 7 days, a sterile cellulose film was placed approximately 1 mm in front of the fungal colony. The fungal colony was allowed to grow for another 7 days, after which the first few millimeters of the cellulose film were colonized by the fungus. To terminate the experiment, fungal hyphae that connected the agar plate with the cellulose film were severed using a scalpel. The film with mycelia was then removed from the agar plate and dried in a laminar airflow cabinet for 30 minutes. After drying, fungal cells were imaged with both IR microscopes sequentially.

### IR microspectroscopy

Conventional IR microspectroscopy was conducted on a Hyperion 3000 IR microscope (Bruker, Billerica, Massachusetts, USA), which was coupled to a Tensor 27 spectrometer (Bruker). The microscope was operated in reflection mode. Hyperspectral images (images in which each pixel contains a full IR spectrum) were collected using a liquid nitrogen-cooled Focal Plane Array detector with 64 by 64 detector elements. The distance between detector elements (and thus the pixel size of hyperspectral images) was approximately 2.3 µm. A Cassegrain objective with a numerical aperture of 0.4 was used, resulting in a spatial resolution of 16.9 µm at 900 cm^−1^ and 8.5 µm at 1,800 cm^−1^. For each pixel, 1,024 scans were co-averaged.

O-PTIR microspectroscopy was conducted on a mIRage system (Photothermal Spectroscopy Corp., Santa Barbara, California, USA). Hyperspectral images were collected in reflection mode using an Avalanche Photodiode Detector (Photothermal Spectroscopy Corp.). IR light was generated by a set of four QCL chips, covering the wavelengths from 770 cm^−1^ to 1,802 cm^−1^. The IR light was directed onto the sample through a set of mirrors in the microscope. Frequencies of IR light that matched the vibrational frequencies of molecules in the sample excited these molecular vibrations, causing thermal expansions in the sample and the formation of a photothermal lens (Fig. 1B) (10). These thermal expansions were probed by a green laser at a wavelength of 532 nm, which was made coaxial and confocal with the IR laser light (Fig. 1B). The thermal expansions of the sample created changes in the intensity of the reflected and scattered green laser light (11). These changes in intensity in the green laser light are proportional to the IR radiation and were used to construct IR spectra (11). Hence, in the case of O-PTIR, the IR light illuminated a large area of the sample, as was the case for conventional IR microspectroscopy, but only the part of the photothermal lens that is probed by the green probe laser (a Cassegrain objective with a numerical aperture of 0.78 was used in this system, resulting in a spatial resolution of 416 nm) contributed to spectral data acquisition in O-PTIR. The pixel size for this technique was 500 nm. For each pixel, three scans were co-averaged.

Laser intensities for both the QCL and green probe laser were set to power levels that did not cause any visible discoloration in the cellulose films or changes in the IR spectra. Discoloration of the cellulose films or changes in IR spectra due to high laser intensities can indicate burning of the sample. These power levels were experimentally determined on cellulose films that were not colonized by fungal cultures. Laser intensities can vary from machine to machine, and different substrates have different sensitivities to laser damage. Hence, laser settings need to be determined experimentally for each microscope and sample case by case. The Globar IR source used in the conventional IR microspectroscopy method emits light of sufficient intensity to cause damage to biological samples, even for prolonged periods (e.g., hours) of exposure to the sample.

### Hyperspectral image analysis

Hyperspectral images collected with both microscopes were analyzed using OCTAVVS version 0.1.17 (https://pypi.org/project/octavvs/; 12). Using OCTAVVS, spectra were corrected for contributions from atmospheric H_2_O and CO_2_ gasses, cut from 900 cm^−1^ to 1,800 cm^−1^, and baseline corrected using a rubberband baseline correction. Spectra were normalized using a cellulose peak at 1,370 cm^−1^. The distribution of chemical compound classes in each hyperspectral image was analyzed using the SIMPLe-to-use Interactive Self-modelling Mixture Analysis (SIMPLISMA) algorithm (13, 14) and Multivariate Curve Resolution with Alternating Least Squares optimization (MCR-ALS) (14) implemented in OCTAVVS. Using these calculated spectral components and their contributions, hyperspectral image pixels were then clustered using *k*-means clustering with three clusters, based on chemical similarity. This number of clusters was selected as it was the smallest number of clusters required to locate the hyphae, their surrounding decomposition zones, and the un-decomposed background cellulose films.

## RESULTS

**Fig 2:**
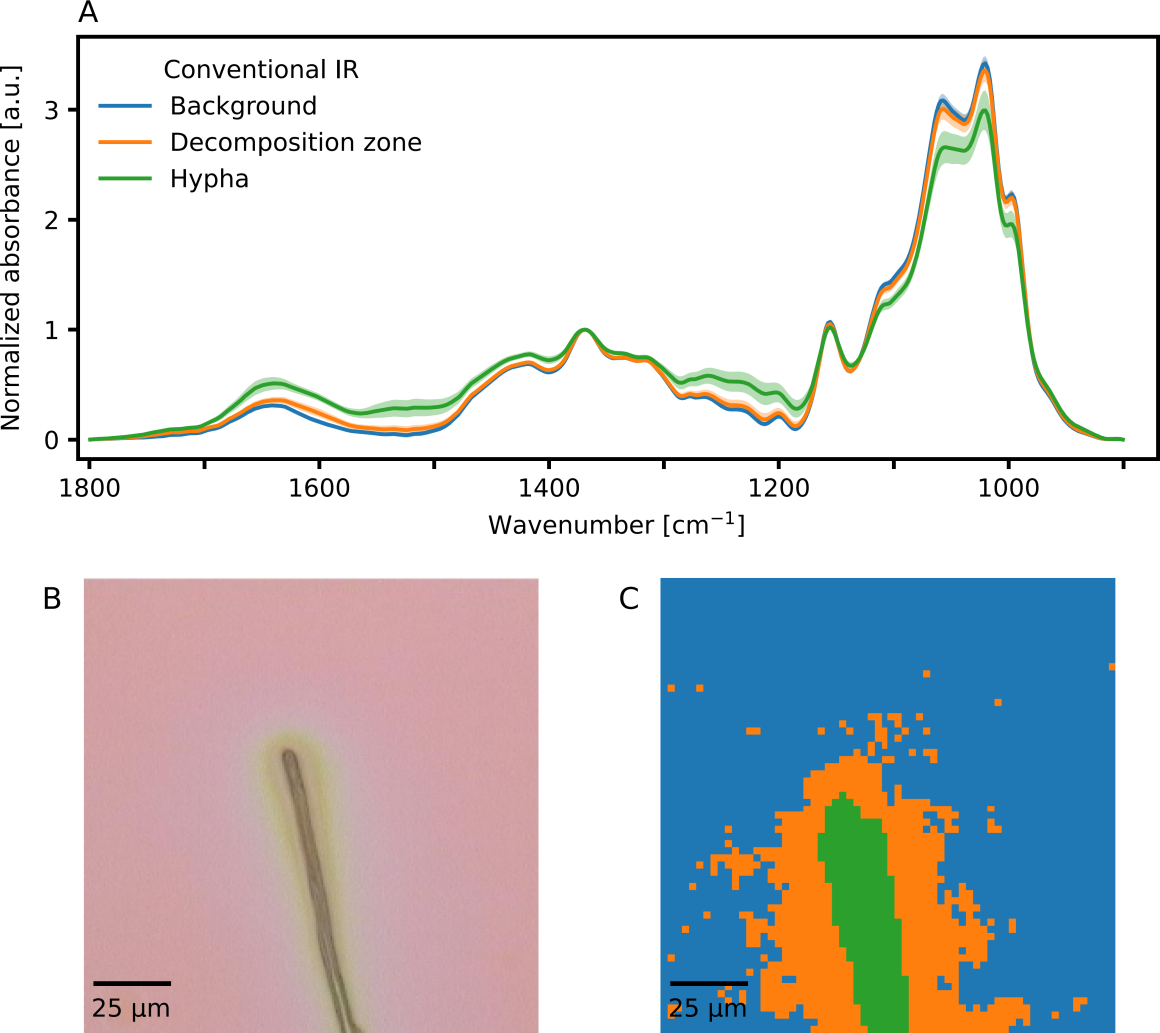
IR imaging results of a hypha of the ectomycorrhizal fungus *Paxillus involutus* growing on a cellulose film collected with a conventional IR microscope. (A) Average IR absorption spectra of pixels clustered to the background (blue), the hypha (green), or the decomposition zone around the hypha (orange). Average spectra are shown ±1 standard deviation. (B) Visible light image of the investigated hypha of *P. involutus*. (C) Cluster map of the IR image collected using conventional IR microspectroscopy. Blue pixels correspond to the undecomposed cellulose film (background). Orange pixels correspond to the decomposition zone that surrounds the hypha. Green pixels correspond to the location of the hypha. Individual spectra were recorded as 1,024 co-averaged scans per pixel (pixel size was 2.3 µm).

MCR-ALS processing and clustering of a hyperspectral image of a hyphal tip of *P. involutus* growing on a cellulose film collected with conventional IR microspectroscopy revealed two distinct zones where the hypha had slightly altered the chemical composition of the cellulose film. A first zone occurred where the hypha itself was located. This zone was approximately 4.6 µm wide and corresponded to the width of the hypha in the white light image. The average IR spectrum of this zone is designated as “Hypha” in Fig. 2A and is marked in green; the spatial distribution of pixels corresponding to this cluster can be seen in Fig. 2C, colored in green. This first zone was surrounded by a second zone that corresponds to the extracellular space of the hypha (the average spectrum that corresponds to this zone was designated as “Decomposition zone” in Fig. 2A in orange, with the distribution of pixels displayed in Fig. 2C in orange). This zone extended approximately 11.1 µm away from the edge of the hypha on both sides of the hypha. In Fig. 2A, the blue spectrum designated as “Background” corresponds to the average cellulose spectrum unaffected by fungal decomposition. The corresponding pixels are displayed in blue in Fig. 2C. A visible light image of the studied cell can be seen in Fig. 2B.

**Fig 3:**
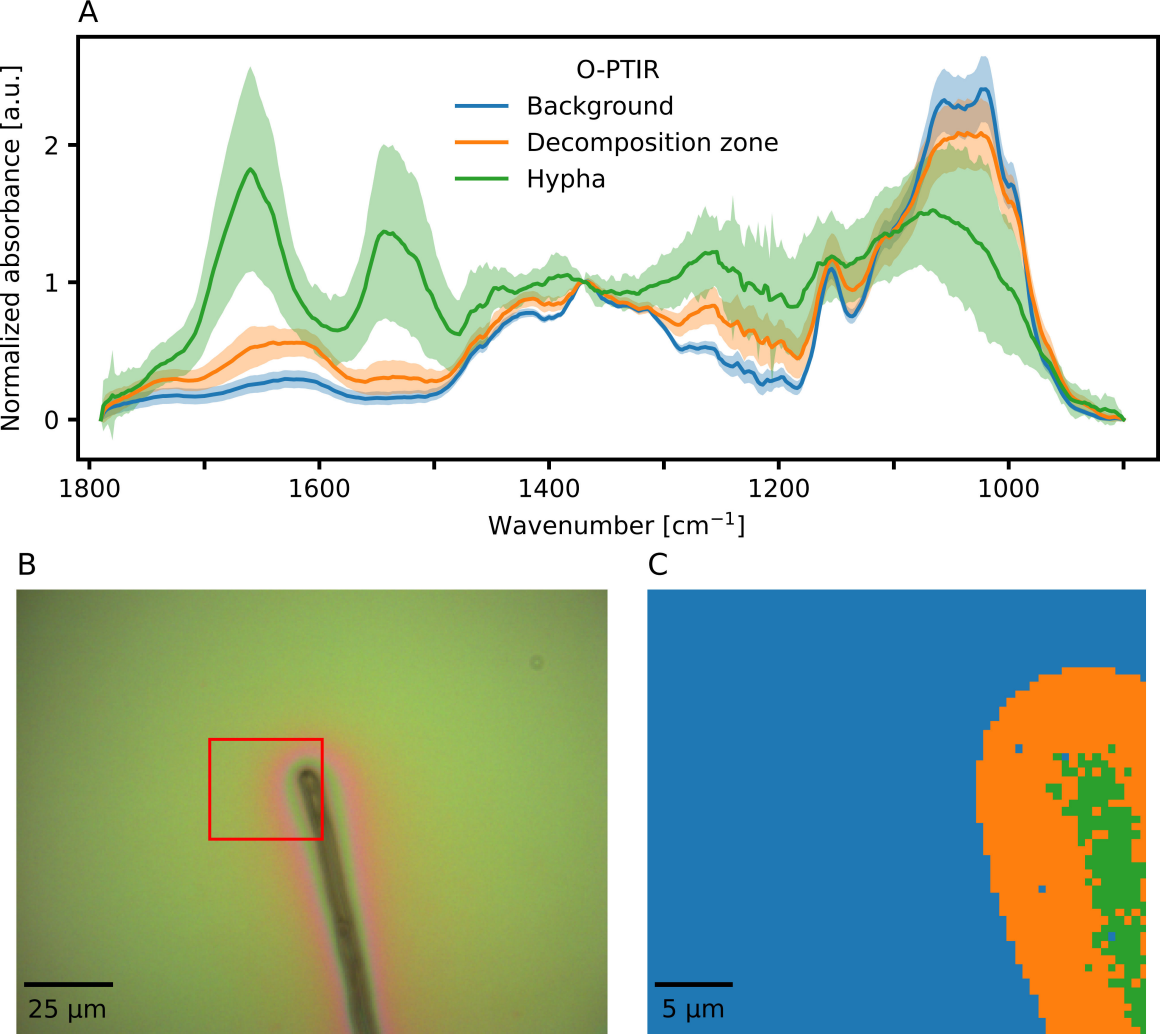
IR imaging results of a hypha of the ectomycorrhizal fungus *Paxillus involutus* growing on a cellulose film collected with O-PTIR microspectroscopy. It is the same hypha that was imaged with conventional IR microspectroscopy, shown in Fig. 2. (A) Average IR spectrum of pixels clustered to the background (blue), the hypha (green), or the decomposition zone (orange). (B) Visible light image of the investigated hypha. Average spectra are shown as ±1 standard deviation. The red rectangle indicates the zone where the IR image was collected from and corresponds to Fig. 3C. (C) Cluster map of the IR image collected using O-PTIR. Blue pixels correspond to the original cellulose film (background). Orange pixels correspond to the decomposition zone. Green pixels correspond to the position of the hypha. This IR cluster map corresponds to the red rectangle displayed in Fig. 3B. Individual spectra were recorded as three co-averaged scans per pixel (pixel size was 500 nm).

MCR-ALS processing and clustering of a hyperspectral image of the same hyphal tip of *P. involutus* collected with O-PTIR revealed a similar zone corresponding to the position of the hypha with a width of approximately 4.6 µm (green spectrum in Fig. 3A and green pixels in Fig. 3C) and a surrounding decomposition zone that extended approximately 6.3 µm away from the edge of the hypha (orange spectrum in Fig. 3A and orange pixels in Fig. 3C). IR spectra unaffected by fungal decomposition are displayed in blue in Fig. 3A and C. A visible light image of the investigated cell, collected with the O-PTIR instrument, is shown in Fig. 3B. The red rectangle in Fig. 3B indicates the zone that was imaged with O-PTIR and corresponds to the size of the IR cluster image in Fig. 3C.

The IR spectra collected using conventional IR microspectroscopy (Fig. 2A) closely resemble each other. A decrease in IR absorption in the carbohydrate region from approximately 1,100 cm^−1^ to 950 cm^−1^ can be seen, with the lowest IR absorption occurring where the hypha is located (green spectrum in Fig. 2A and green pixels in Fig. 2C) and the highest absorption for the background (blue spectrum in Fig. 2A and blue pixels in Fig. 2C). The decomposition zone that surrounds the hypha has an intermediate absorption intensity in this wavenumber region, in between that of the hypha and the background (orange spectrum in Fig. 2A and orange pixels in Fig. 2C). Conversely, the IR absorption between 1,700 cm^−1^ and 1,600 cm^−1^ is lowest for the background and highest for the hyphal tip, with the decomposition zone being intermediary to these two zones. IR absorption in this wavenumber region can be ascribed to C=O vibrations that could result from the oxidation of cellulose (15), but also to the presence of peptide bonds in proteins inside the fungal hypha (16). Therefore, the observed increase in absorbance in this wavenumber region could be caused by the appearance of intracellular proteins when measuring closer to the fungal hypha or by increased oxidation of the cellulose closer to the hyphae. Due to the limited spatial resolution of conventional IR microspectroscopy, it is difficult to ascribe increases in IR absorption in this wavenumber region to either one of these explanations unequivocally.

In the O-PTIR spectra in Fig. 3A, the IR signal in the 1,100 cm^−1^ to 950 cm^−1^ wavenumber region decreases the closer data are collected to the hypha. However, distinct changes in the spectra within this carbohydrate region can now be observed that were not apparent in the IR spectra collected with conventional IR microspectroscopy (Fig. 2A). In the case of O-PTIR data, the IR signal in the wavenumber region between 1,700 cm^−1^ and 1,600 cm^−1^ is clearly higher in the average spectrum collected from the decomposition zone (orange spectrum in Fig. 3A) than it is in the average spectrum collected from the background (blue spectrum in Fig. 3A). A further increase in the IR signal at the position where the hypha is located (green pixels in Fig. 3C) is masked by the clear presence of two new strong absorption bands at regions between 1,650 cm^−1^ and 1,550 cm^−1^. These two new bands correspond to the absorption bands of C=O and C-N/N-H, respectively, in peptide bonds in proteins (16). Thanks to the high spatial resolution of O-PTIR, these proteins can be precisely localized to the intracellular space of the hypha in Fig. 3. The increase in IR absorption in the 1,700 cm^−1^ to 1,600 cm^−1^ due to the oxidation of cellulose can thus be clearly distinguished from the contribution of C=O from peptide bonds of intracellular proteins, making it possible to conclude that the cellulose has indeed been oxidized by *P. involutus*. This conclusion could not be unequivocally drawn from the data collected using conventional IR microspectroscopy.

Two additional hyphae of *P. involutus* growing on cellulose were examined using conventional IR and O-PTIR microspectroscopy sequentially (Fig. S2 to S5). The obtained data closely resemble those reported in Fig. 2 and 3.

**Fig 4:**
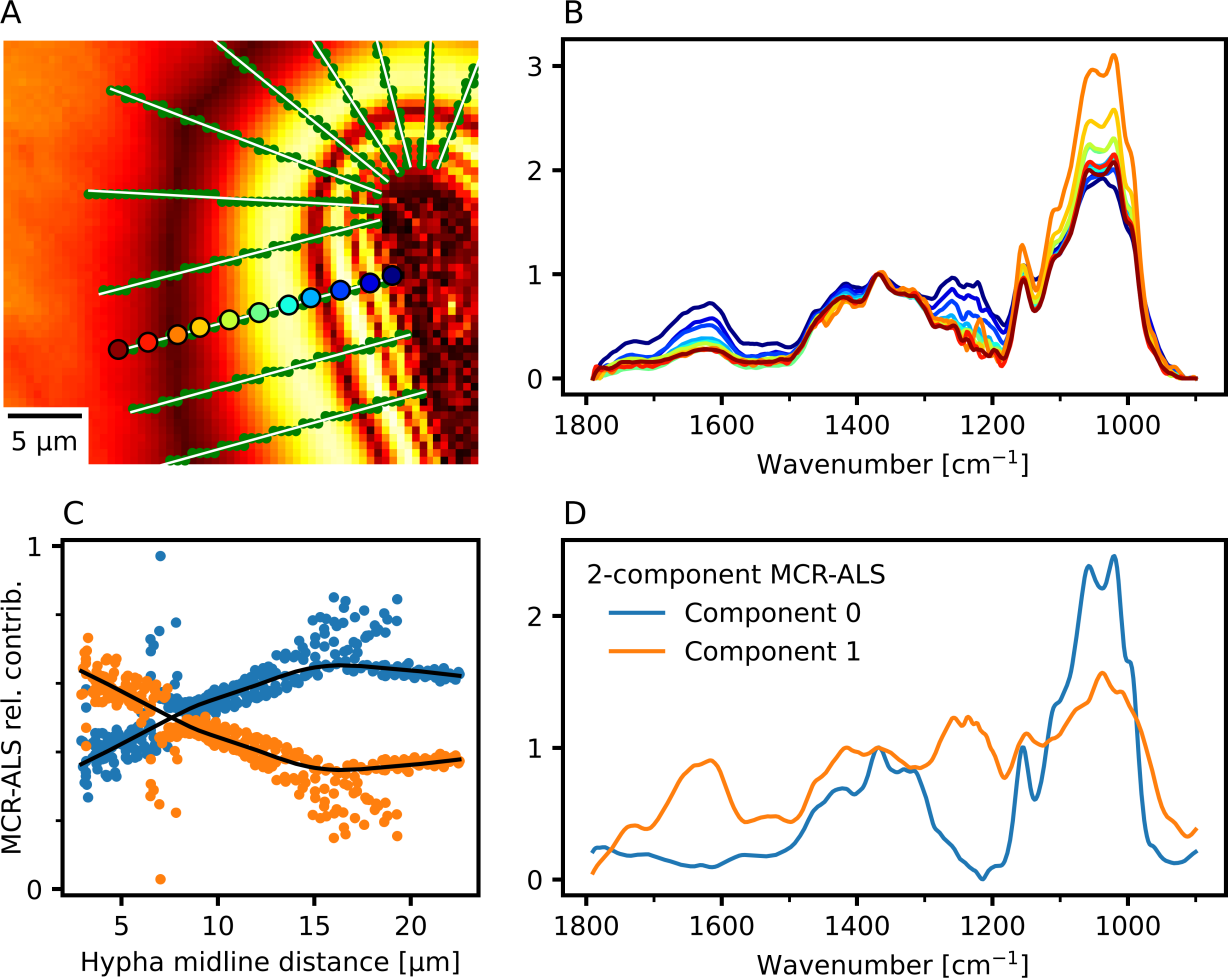
Gradient of spectral compositions across the decomposition zone. (A) Heatmap of average absorbance values in the O-PTIR image. Selected spectra (green dots) were examined along radial cuts (white) through the decomposition zone. (B) Baseline corrected and normalized spectra from 11 spectra along one radial cut (filled circles in A) showing typical differences in the decomposition zone from the unaffected cellulose film (dark red) to near the hypha (blue). (C–D) MCR-ALS decomposition of the normalized selected spectra into two non-negative components. (C) The contributions of the two spectral components (blue and orange) for each spectrum, as functions of the distance to the center line of the hypha. Lowess regression (black lines) shows a steady change in the chemical composition with distance from the hypha in the decomposition zone. The contributions follow this trend closely, deviating mainly because of a low-intensity band at 14 µm from the hyphal center. (D) The corresponding spectral components (blue and orange). Individual spectra were recorded as three co-averaged scans per pixel. Pixel size was 500 nm.

The high spatial resolution and spectral quality of the O-PTIR data encourage a closer look at gradual changes in a region of interest. Figure 4A illustrates the selection of spectra in the decomposition zone: a set of radial lines (white) were drawn from the approximate edge of the hypha, and pixels (338 green dots) near the lines were selected for further analysis. Examples of individual spectra along one of the radial lines (colored points in Fig. 4A) are shown in Fig. 4B, suggesting a gradual change in the spectral regions of interest. The 338 selected spectra were analyzed using MCR-ALS with two components, to find the two major contributions to the spectra and their relation to the position within the decomposition zone. The contribution of one of the components (orange points, Fig. 4C) increased linearly with the distance to the midline of the hypha but was constant outside the decomposition zone. This MCR-ALS component carries the signs of decomposition, e.g., the IR signal from the C = O region (orange line in Fig. 4D), while the other component (blue points and line) appears to represent the unaltered cellulose. Note, however, that the background consists of a mixture of the two spectral components. The seemingly pure examples of the two components about 7 µm from the hyphal midline (Fig. 4C) may just be noise from spectra with very low intensities (dark bands in Fig. 4A).

**Fig 5:**
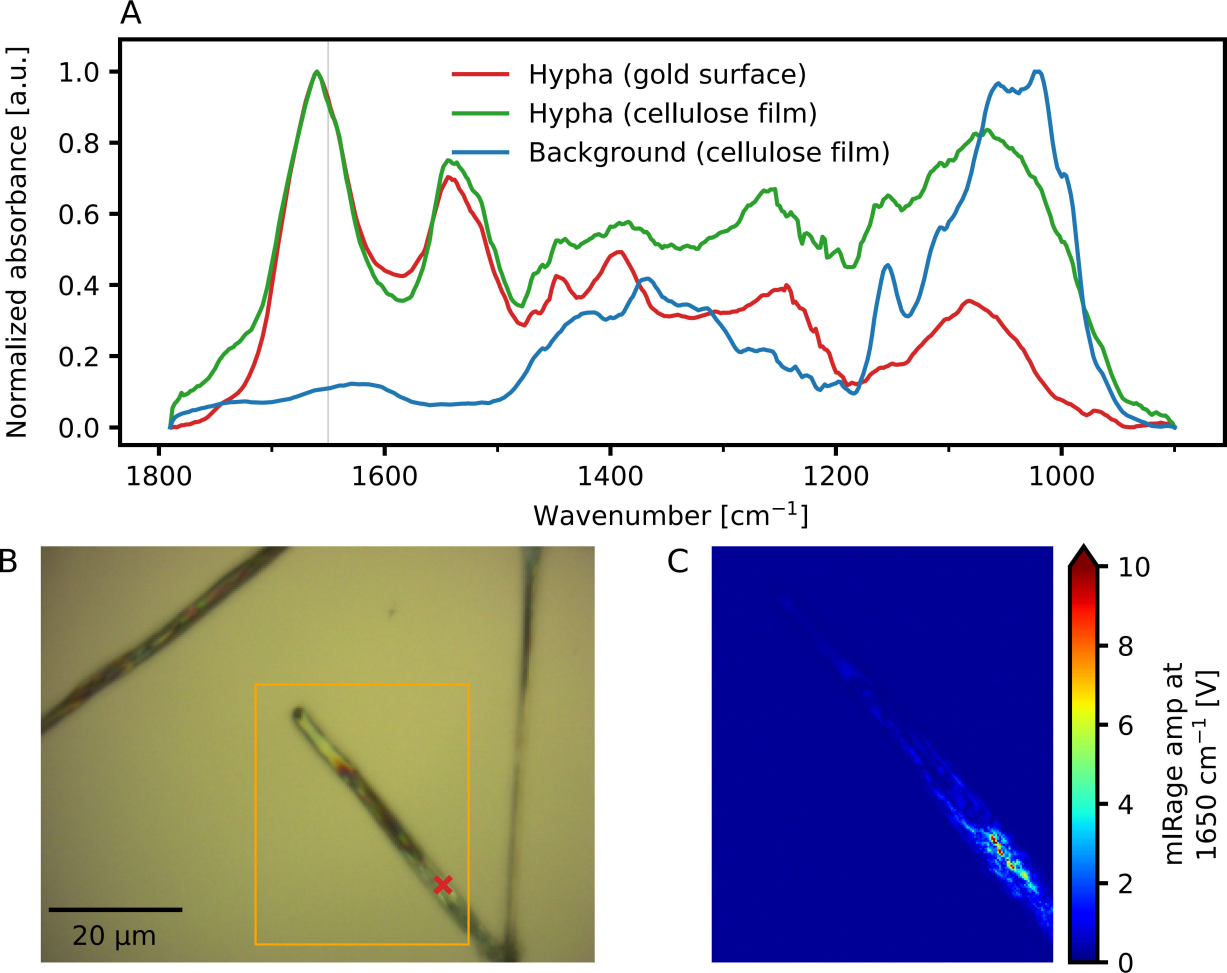
O-PTIR imaging of a single cell of the ectomycorrhizal fungus *Paxillus involutus* growing on a bare gold-coated microscope slide without a cellulose film. (A) The red spectrum is a single spectrum collected using O-PTIR on the hypha of *P. involutus* growing on a bare gold-coated microscope slide. The position this spectrum was collected from is indicated with a red mark in panel (B). The blue spectrum is the background cellulose spectrum shown in Fig. 3A, added to this figure for comparison. The green spectrum is a single O-PTIR spectrum collected on the hypha that was growing on the cellulose film from Fig. 3. Note the similarity between the green and the red spectra. In both cases (the hypha growing on gold, red spectrum, and the hypha growing on a cellulose film, green spectrum), mostly intracellular proteins from the fungal cell are detected. (B) Visible light image of a cell of *P. involutus* growing on a bare gold-coated microscope slide. The orange rectangle corresponds to the position from which a single wavenumber IR image was collected, displayed in panel C. (C) IR single wavenumber image collected at 1,650 cm^−1^, showing the distribution of proteins inside the *P. involutus* cell. The spectrum collected on the cell of *P. involutus* growing on gold was recorded as three co-averaged scans. The pixel size in Fig. 5C is 100 nm.

To further illustrate that the chemical changes observed in the cellulose film did not result from the presence of cellular components in the hypha, an IR spectrum from proteins located in a cell of *P. involutus* growing on a gold-coated microscope slide without a cellulose film was recorded (red spectrum in Fig. 5A) compared to an IR spectrum from the cellulose background film (blue spectrum in Fig. 5A). The IR spectrum of proteins located in a hyphal tip of *P. involutus* growing on a cellulose film was included for comparison (green spectrum in Fig. 5A). The blue spectrum in Fig. 5A is the same spectrum presented in Fig. 3A. In the cellulose spectrum (blue spectrum in Fig. 5), the strongest IR absorption occurs in the wavenumber region from 1,100 cm^−1^ to 950 cm^−1^. This IR absorption is attributed to C-O stretching vibrations in cellulose (17). The other clear peak in the blue spectrum in Fig. 5A at 1,160 cm^−1^ can be attributed to C-O-C ether stretching in cellulose (18). The IR spectra collected from hyphal tips growing on gold (red spectrum in Fig. 5A) or growing on the cellulose film (green spectrum in Fig. 5A) lack the strong absorption bands in the 1,200 cm^−1^ to 950 cm^−1^ wavenumber region observed in cellulose. On the other hand, the IR spectra collected from proteins inside hyphal tips show two strong absorption bands in the 1,700 cm^−1^ to 1,500 cm^−1^ wavenumber region. The absorption band around 1,650 cm^−1^ can be primarily attributed to C=O stretching vibrations, and the absorption band at 1,550 cm^−1^ can be attributed to C-N and N-H bending vibrations in the peptide bonds of proteins (16). Since the IR absorption bands of cellulose and those of the intracellular proteins in hyphae are separated from each other by at least 300 cm^−1^, we conclude that the chemical changes observed in cellulose cannot be the result of spectral contributions from the fungal cell and can only be attributed to extracellular oxidation of the cellulose film by the fungus.

## DISCUSSION

Comparing the results obtained using both conventional and O-PTIR microspectroscopy, we show that the spatial resolution at which microbial decomposition is studied can drastically affect our interpretation of the ability of microorganisms to decompose organic compounds. Data collected using conventional IR microspectroscopy suggested that *P. involutus* has only a very limited ability to alter the chemical structure of cellulose. O-PTIR data, on the other hand, indicated that *P. involutus* can drastically alter the structure of cellulose. If one thinks of the volume of a sample that is probed by conventional IR microspectroscopy (Fig. 1) and O-PTIR (Fig. 2), conventional IR microspectroscopy can be thought of as a less spatially sensitive technique compared to O-PTIR. In that light, analytical techniques with lower spatial resolution will average out larger volumes of a sample, making a sample look more homogeneous than it really is. For many purposes, this is acceptable and appropriate. However, when studying processes that are very localized, such as the decomposition of organic matter by microorganisms, these processes clearly need to be studied at the appropriate spatial scales. This way, more correct conclusions about the chemical changes that are introduced in organic matter can be drawn, and the ability of microorganisms to decompose different organic substances can be more accurately evaluated. This observation thus implies that the ability of many microorganisms to decompose organic matter may have been underestimated in past studies where more bulk approaches have been used.

A downside to O-PTIR is the need for the QCL to cycle through the range of wavelengths of interest and raster scan through points within an image, making the collection of full hyperspectral images much slower than in conventional IR microspectroscopy (the Globar IR source sends out IR light with a wide range of wavelengths simultaneously, while a focal plane array detector detects IR light at all points in an image simultaneously). Hence, conventional IR microspectroscopy and O-PTIR are highly complementary techniques. For example, conventional IR microspectroscopy could be used to obtain a broad overview of the distribution of chemical compound classes in a sample for which little prior information is available, which can lead to the identification of wavenumbers of interest for that sample. Images can then be collected for these selected wavenumbers with high spatial resolution using O-PTIR. Finally, a limited number of spectra can be collected from regions of interest in a sample to make more detailed comparisons of the chemical makeup of a sample.

The extent to which ectomycorrhizal fungi decompose soil organic matter and the mechanisms by which they do so are highly debated. Ectomycorrhizal fungi have lost many of the genes encoding plant cell-wall degrading enzymes following adaptation to a symbiotic lifestyle (19, 20). This is relevant to soil organic matter decomposition because the major constituents of soil organic matter are compounds derived from plant cell walls, including lignocellulose. We have shown in the past that *P. involutus* has the capacity to decompose soil organic matter using a non-enzymatic, oxidative mechanism involving the action of reactive oxygen species generated through an electron shuttle-mediated Fenton reaction, similar to that of brown-rot wood-decaying fungi (5, 6). Using conventional IR microspectroscopy, we have previously shown that *P. involutus* can decompose lignin through oxidation (21). Data collected using conventional IR microspectroscopy in this study indicated that *P. involutus* has a very limited ability to decompose cellulose. However, the higher spatial resolution of the O-PTIR method showed that *P. involutus* has a substantial capacity to decompose cellulose using oxidative mechanisms. The mechanisms underlying the oxidation of cellulose are not known but can involve the action of reactive oxygen species as described in brown-rot fungi (22). In addition, *P. involutus* has several genes that encode lytic polysaccharide monooxygenases (6), which are enzymes that can catalyze the oxidative cleavage of cellulose (23). Moreover, we demonstrate that O-PTIR microspectroscopy has a resolution that will allow studies of the chemical structure of the decomposition zone in more detail. Most likely, this microenvironment contains extracellular polymeric substances secreted by fungi (21). Extracellular polymeric substance matrices have been thought to play an important function in regulating extracellular decomposition reactions in fungi, but their structure and properties are not well known (24). The high spatial resolution and chemical sensitivity of O-PTIR will be a valuable tool to further investigate the involvement of extracellular polymeric substance matrices in fungal decomposition processes.

In conclusion, O-PTIR is a label-free and non-destructive technique that allows the spatial distribution of chemical compound classes to be determined at sub-micrometer spatial scales, while samples can be held under ambient conditions, opening up the possibility of studying the interactions between microorganisms and their direct surroundings *in situ*.

## Data Availability

The datasets generated in this work can be retrieved from the authors upon reasonable request.

## SUPPLEMENTAL MATERIAL

Supplemental material aem.01489-23-s0001.docx Supplemental figures that show IR data of the deacetylation of cellulose films and two additional hyphal tips that were analyzed.

ASM does not own the copyrights to Supplemental Material that may be linked to, or accessed through, an article. The authors have granted ASM a non-exclusive, world-wide license to publish the Supplemental Material files. Please contact the corresponding author directly for reuse.
