# Supplementary material for: Elucidating fungal decomposition of organic matter at sub-micrometer spatial scales using optical photothermal infrared (O-PTIR) microspectroscopy
Source: Appl Environ Microbiol. 2024 Jan 30;90(2):e01489-23. doi: 10.1128/aem.01489-23 (PMC10880621; doi:10.1128/aem.01489-23)
Supplement: Supplemental material — Supplemental figures that show IR data of the deacetylation of cellulose films and two additional hyphal tips that were analyzed. [file aem.01489-23-s0001.docx]

Supplemental Files

Elucidating fungal decomposition of organic matter at sub-micrometer spatial scales using optical photothermal infrared (O-PTIR) microspectroscopy

Michiel Op De Beeck*, Carl Troein, Carsten Peterson, Anders Tunlid, Per Persson

*** Correspondence:** Michiel Op De Beeck: michiel.op_de_beeck@cec.lu.se

# Supplemental Figures


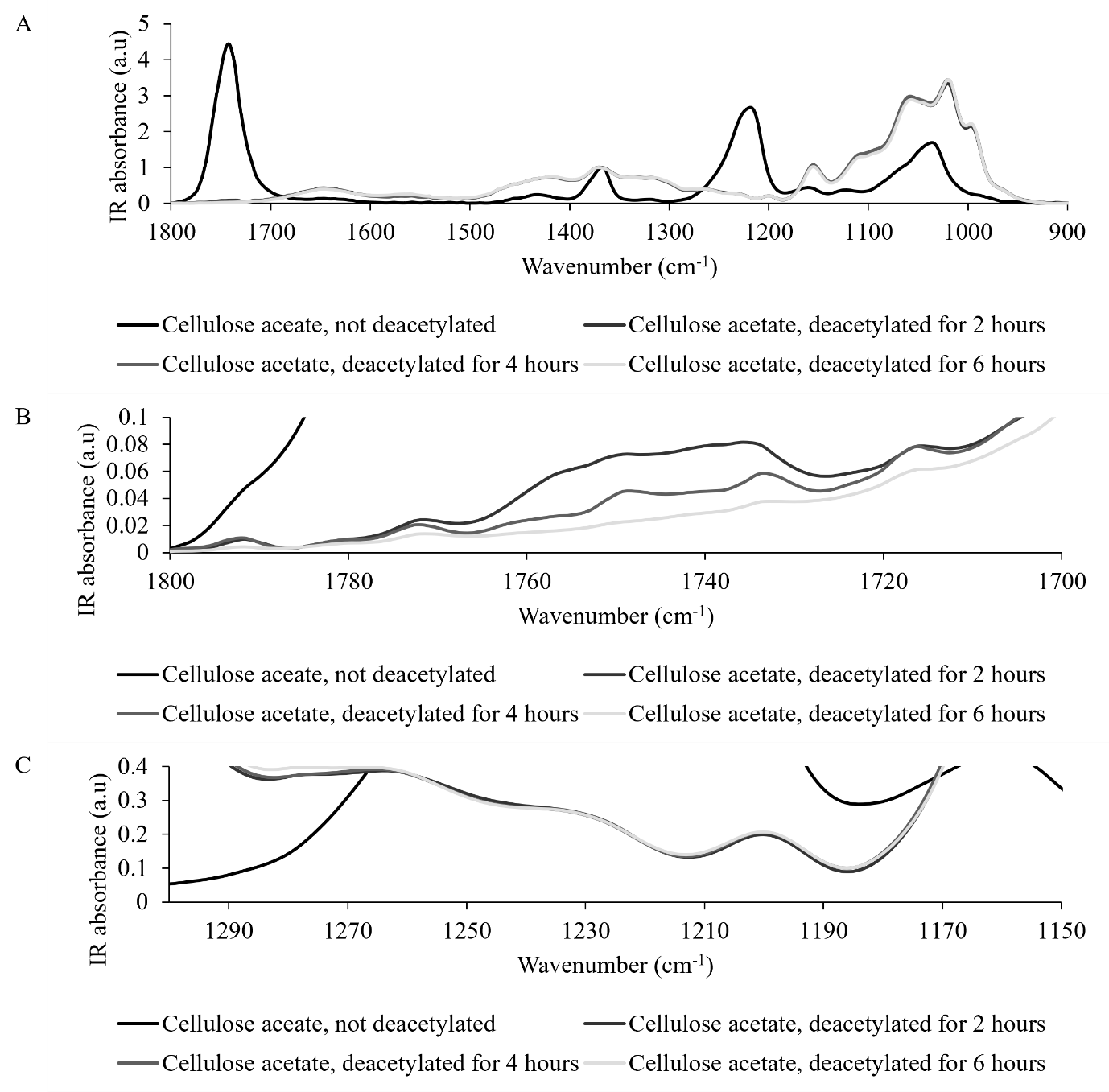


**Supplemental Figure S1.** IR spectra of cellulose acetate films during deacetylation. Spin-coated cellulose acetate films were deacetylated using 0.1 M NaOH for 2, 4 and 6 hours to remove the acetyl groups, resulting in regenerated cellulose films. Further details of the preparation of the regenerated cellulose films can be found in the Materials and Methods section in the main text. All spectra were recorded with a conventional IR microscope, as detailed in the Materials and Methods section in the main text. IR spectra represent averages of 50 randomly selected points in hyperspectral images of regenerated cellulose films. IR spectra were cut from 1800 cm^-1^ to 900 cm^-1^, rubberband baseline corrected and normalized to the 1370 cm^-1^ cellulose peak. IR absorbance values are given in arbitrary units (a.u.). A. Overview of IR spectra from 1800 cm^-1^ to 900 cm^-1^. B. Close-up of the acetate peak at 1742 cm^-1^. Note that the acetate peak is completely removed after 6 hours of deacetylation. C. Close-up of the acetate peak at 1219 cm^-1^. Note that the acetate peak can no longer be detected after 2 hours of deacetylation.

**Supplemental Figure S2.** IR imaging results of a hypha of the ectomycorrhizal fungus *Paxillus involutus* growing on a cellulose film collected with a conventional IR microscope. The hypha imaged in Supplemental Fig. S2 and Supplemental Fig. S3 is the same cell. A. Average IR absorption spectra of pixels clustered to the background (blue), the hypha (green) or the decomposition zone around the hypha (orange). Average spectra are shown +/- one standard deviation. B. Visible light image of the investigated hypha of *P. involutus*. C. Cluster map of the IR image collected with conventional IR microspectroscopy. Blue pixels correspond to the undecomposed cellulose film (background). Orange pixels correspond to the decomposition zone that surrounds the hypha. Green pixels correspond to the location of the hypha. Individual spectra were recorded as 1024 co-averaged scans per pixel. Pixel size is 2.3 µm.

**Supplemental Figure S3.** IR imaging results of a hypha of the ectomycorrhizal fungus *Paxillus involutus* growing on a cellulose film collected with Optical Photothermal Infrared (O-PTIR) microspectroscopy. The hypha imaged in Supplemental Fig. S2 and Supplemental Fig. S3 is the same cell. A. Average IR spectrum of pixels clustered to the background (blue), the hypha (green) or the decomposition zone (orange). B. Visible light image of the investigated hypha. Average spectra are shown +/- one standard deviation. The red rectangle indicates the zone where the IR image was collected from and corresponds to the size of Supplemental Fig. S3C. C. Cluster map of the IR image collected with O-PTIR. Blue pixels correspond to the original cellulose film (background). Orange pixels correspond to the decomposition zone. Green pixels correspond to the position of the hypha. This IR cluster map corresponds to the red rectangle displayed in Supplemental Fig. S3B. Individual spectra were recorded as 3 co-averaged scans per pixel. Pixel size is 1 µm.

**Supplemental Figure S4.** IR imaging results of a hypha of the ectomycorrhizal fungus *Paxillus involutus* growing on a cellulose film collected with a conventional IR microscope. The hypha imaged in Supplemental Fig. S4 and Supplemental Fig. S5 is the same cell. A. Average IR absorption spectra of pixels clustered to the background (blue), the hypha (green) or the decomposition zone around the hypha (orange). Average spectra are shown +/- one standard deviation. B. Visible light image of the investigated hypha of *P. involutus*. C. Cluster map of the IR image collected with conventional IR microspectroscopy. Blue pixels correspond to the undecomposed cellulose film (background). Orange pixels correspond to the decomposition zone that surround the hypha. Green pixels correspond to the location of the hypha. Individual spectra were recorded as 1024 co-averaged scans per pixel. Pixel size is 2.3 µm.

**Supplemental Figure S5.** IR imaging results of a hypha of the ectomycorrhizal fungus *Paxillus involutus* growing on a cellulose film collected with Optical Photothermal Infrared (O-PTIR) microspectroscopy. The hypha imaged in Supplemental Fig. S4 and Supplemental Fig. S5 is the same cell. A. Average IR spectrum of pixels clustered to the background (blue), the hypha (green) or the decomposition zone (orange). B. Visible light image of the investigated hypha. Average spectra are shown +/- one standard deviation. The red rectangle indicates the zone where the IR image was collected from and corresponds to the size of Supplemental Fig. S5C. C. Cluster map of the IR image collected with O-PTIR. Blue pixels correspond to the original cellulose film (background). Orange pixels correspond to the decomposition zone. Green pixels correspond to the position of the hypha. This IR cluster map corresponds to the red rectangle displayed in Supplemental Fig. S5B. Individual spectra were recorded as 3 co-averaged scans per pixel. Pixel size is 1 µm.
